# Formal Security Reassessment of the 5G-AKA-FS Protocol: Methodological Corrections and Augmented Verification Techniques

**DOI:** 10.3390/s24247979

**Published:** 2024-12-13

**Authors:** Yongho Ko, I Wayan Adi Juliawan Pawana, Ilsun You

**Affiliations:** 1 Department of Financial Information Security, Kookmin University, Seoul 02707, Republic of Korea; koyh0911@kookmin.ac.kr (Y.K.); adijuliawanpawana@unud.ac.id (I.W.A.J.P.); 2 Department of Electrical Engineering, Udayana University, Bali 80361, Indonesia

**Keywords:** 5G security, forward secrecy (FS), 5G-AKA-FS, ProVerif

## Abstract

The 5G-AKA protocol, a foundational component for 5G network authentication, has been found vulnerable to various security threats, including linkability attacks that compromise user privacy. To address these vulnerabilities, we previously proposed the 5G-AKA-Forward Secrecy (5G-AKA-FS) protocol, which introduces an ephemeral key pair within the home network (HN) to support forward secrecy and prevent linkability attacks. However, a re-evaluation uncovered minor errors in the initial BAN-logic verification and highlighted the need for more rigorous security validation using formal methods. In this paper, we correct the BAN-logic verification and advance the formal security analysis by applying an extended SVO logic, which was adopted as it provides a higher level of verification compared to BAN logic, incorporating a new axiom specifically for forward secrecy. Additionally, we enhance the ProVerif analysis by employing a stronger adversarial model. These refinements in formal verification validate the security and reliability of 5G-AKA-FS, ensuring its resilience against advanced attacks. Our findings offer a comprehensive reference for future security protocol verification in 5G networks

## 1. Introduction

As mobile communication technologies rapidly evolve, the need for secure and privacy-preserving authentication in 5G networks becomes increasingly critical. The 5G-AKA protocol [1], the primary authentication mechanism in 5G networks, has demonstrated susceptibility to security risks, including linkability attacks, which compromise user privacy. The introduction of 3GPP’s Authentication and Key Management for Applications (AKMA) standard [2] further underscores the necessity for an upgraded 5G-AKA protocol that achieves forward secrecy while ensuring user unlinkability. Although our previous work proposed 5G-AKA-FS [3] to mitigate these issues through ephemeral key generation within the home network (HN), recent analysis identified minor errors in the BAN-logic [4] verification and highlighted the need for an enhanced ProVerif [5] evaluation to comprehensively validate its security.

### 1.1. Related Works

The evolution of cellular networks from 4G to 5G has brought significant advancements in security and privacy to address the growing complexity and sensitivity of applications and services. Its primary authentication protocols are central to the 5G security framework: the 5G Authentication and Key Agreement (5G-AKA) and the Extensible Authentication Protocol-Authentication and Key Agreement (EAP-AKA’). These protocols incorporate enhancements to safeguard communication networks against emerging threats while improving authentication efficiency and performance. Among these enhancements is the inclusion of mechanisms that ensure forward secrecy, a critical property that protects the confidentiality of session keys even if long-term credentials are compromised.

The 5G-AKA’ [6] was designed to address linkability attacks by reusing the $K_{HN}$ key, which is established between a user equipment (UE) device and its corresponding home network (HN) through ECIES, to encrypt the SUPI during the initial phase. Specifically, the protocol enables the HN to substitute its random number within the authentication token (AUTN) with a value encrypted using $K_{HN}$. Relying on the freshness of $K_{HN}$, the UE can utilize the encrypted value to mitigate both replay and linkability attacks. However, the protocol remains susceptible to active attacks by a malicious serving network (SN), as it permits the transmission of the anchor key $K_{SEAF}$ to the SN without first authenticating the UE with the HN. Furthermore, the protocol does not fully support perfect forward secrecy (PFS).

The SUCI-AKA protocol [7] was introduced to achieve PFS for the anchor key $K_{SEAF}$ and to defend against linkability attacks. It utilizes the $K_{HN}$ key, which is originally derived through ECIES, to encrypt the SUPI for the computation of the master session key $K_{AUSF}$. Specifically, because the $K_{HN}$ is considered fresh and trusted by both the UE and the HN, it replaces the sequence number, effectively mitigating linkability attacks. However, if the long-term *K* and the HN’s private key $sk_{HN}$ are compromised, an attacker could reconstruct the anchor key $K_{SEAF}$, thereby compromising the protocol’s PFS.

The 5G-IPAKA [8] was developed to enable mutual authentication between the UE and SN, secure key exchange, and PFS. It aims to achieve PFS by deriving the anchor key using the ECIES-derived secret $K_{HN}$. In this protocol, the HN transmits the anchor key $K_{SEAF}$ to the SN before completing the UE’s authentication. This mechanism allows the UE and the SN to authenticate each other using the Message Authentication Code (MAC) generated and exchanged by both parties with $K_{SEAF}$, thereby achieving mutual authentication. However, the protocol’s PFS is compromised if the long-term key *K* and the HN’s private key $sk_{HN}$ are leaked. Additionally, because the HN can send $K_{SEAF}$ to the SN without verifying the UE’s identity, the protocol is vulnerable to active attacks by malicious SNs. Moreover, deviations from standard procedures introduce compatibility challenges.

The 5GAKA-LCCO [9] was designed to reduce high communication and computation overheads while addressing SUCI replay attacks. The SN initiates communication in this protocol by transmitting the current timestamp and a randomly generated nonce to the UE. The UE uses these values to compute the key block by incorporating the long-term key *K* and the ECIES-derived secret key $k_{HN}$. This key block generation enhances the protocol’s efficiency by optimizing communication and computational overhead. Additionally, using timestamps helps mitigate resource exhaustion and SUCI replay attacks. However, vulnerabilities to PFS arise during the computation of the master session key $K_{AUSF}$, as it depends on the long-term key *K* and the secret key $sk_{HN}$, which could be compromised. Furthermore, the reliance on timestamps introduces a time synchronization requirement, which poses challenges in mobile communication scenarios like roaming. Lastly, deviations from existing standards may lead to compatibility issues. See Table 1.

### 1.2. Contributions

In this paper, we address these limitations and present a robust formal verification of 5G-AKA-FS. The primary contributions of our work are as follows:

- **BAN-logic correction**: We correct minor errors in the initial BAN-logic verification of 5G-AKA-FS, strengthening its foundational security assurances.
- **Enhanced SVO logic application**: To overcome the limitations of BAN logic, we apply SVO logic [10], one of the most advanced modal logic frameworks, extending it with a new axiom specifically designed to support forward secrecy in 5G-AKA-FS.
- **Reinforced ProVerif verification**: We enhance the adversarial model within ProVerif, increasing the rigor of the security analysis by simulating more powerful attack scenarios compared to previous studies.

The remainder of this paper is organized as follows. In Section 2, we present the materials and methods used in this study. Section 3 provides a detailed review of the 5G-AKA-FS protocol, explaining its structure and core security mechanisms. Section 4 describes the enhanced formal security verification process, including corrections to the BAN-logic verification and an improved verification using the expanded SVO logic, followed by a strengthened analysis with ProVerif. Finally, Section 5 concludes the paper.

## 2. Materials and Methods

This paper was developed and refined with the assistance of OpenAI’s ChatGPT (version: GPT-4) [11]. ChatGPT is a large language model developed by OpenAI, designed to understand and generate human-like text using natural language processing technology. In this paper, ChatGPT was employed to draft the initial version, improve sentence structure, and enhance the overall clarity of the manuscript. All final decisions regarding the content were made by the authors.

## 3. 5G-AKA-FS Review

### 3.1. Notation

The explanation of symbols and terms used in the 5G-AKA-FS protocol is shown in Table 2.

### 3.2. 5G-AKA-FS Protocol

The 5G-AKA-FS protocol is an extension of 5G-AKA designed to support forward secrecy and resistance to linkability attacks. The 5G-AKA-FS protocol operates in two phases. In the initial phase, the UE generates an ephemeral public–private key pair $\left(epk_{UE},epk_{UE}^{-1}\right)$ to serve as key materials for the DHK and transmits the SUCI, containing $epk_{UE}$ and encrypted SUPI, secured with a key derived through an ECDH key agreement between the UE’s ephemeral private key $epk_{UE}^{-1}$ and HN’s public key $pk_{HN}$ accompanied by an MAC. The HN decrypts the SUCI with the shared key from an ECDH key agreement using its private key, $sk_{HN}$, and $epk_{UE}$ as key materials. The second phase is a challenge–response phase, during which the UE and HN perform mutual authentication and, in conjunction with the SN, establish the anchor key $K_{SEAF}$. The HN generates an ephemeral public–private key pair $\left(epk_{HN},epk_{HN}^{-1}\right)$ using $epk_{HN}$ as the random challenge RAND. The DHK is then derived through $epk_{HN}^{-1}$ and the UE’s ephemeral public key $epk_{UE}$ as key materials, supporting forward secrecy.

The 5G-AKA-FS protocol is specifically designed to counter linkability attacks. In the original 5G-AKA protocol, the UE relies on the SQN to verify the AUTN-MAC freshness, which causes the protocol to be vulnerable to replay attacks, where an attacker could exploit the Sync_failure message. To address this, 5G-AKA-FS enhances MAC freshness by integrating the DHK, ensuring that each session generates a unique MAC value. This approach guarantees that even if an MAC is retransmitted, a fresh DHK, derived from the UE’s ephemeral public key in the initial phase, allows for earlier detection of any replayed MAC at the MAC verification before reaching the SQN verification.

**Figure 1:**
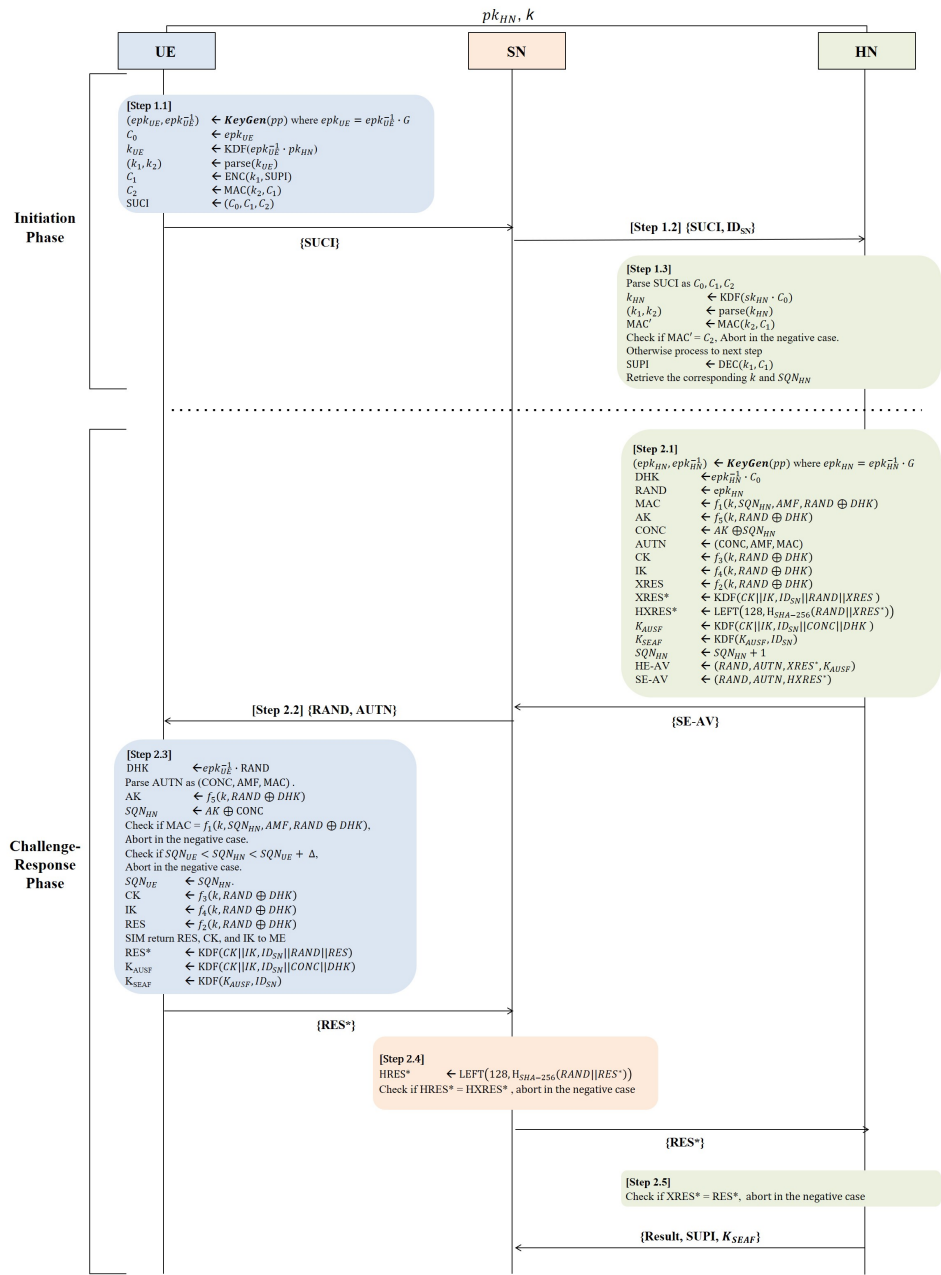
The 5G-AKA-FS protocol.

The description of the 5G-AKA-FS protocol follows, and is shown in Figure 1.

#### 3.2.1. The Initiation Phase: Step 1

In this phase, the UE generates an ephemeral public–private key pair ($epk_{UE},epk_{UE}^{-1}$), to serve as key material for the DHK. Then, the UE transmits SUCI, containing the ephemeral public key $epk_{UE}$ and the encrypted SUPI, secured using both HN’s public key $pk_{HN}$ and the UE’s ephemeral private key $epk_{UE}^{-1}$ as material for the ECDH key agreement and accompanied by an MAC to the SN. Then, the SN forwards both SUCI and its identifier $ID_{SN}$ to the HN. In response, the HN first verifies the MAC sent by the UE. If this verification fails, the process is aborted; otherwise, it proceeds with the de-concealment of the SUCI to retrieve the SUPI using the HN’s private key $sk_{HN}$. This process is shown in Algorithms 1–3.

**Algorithm 1** Initiation Step 1.1 [UE]
   *Inputs:* 
$pk_{HN}$      Generate an ephemeral public-private key pair ($epk_{UE},epk_{UE}^{-1}$)      Compute $C_{0}\leftarrow epk_{UE}$      Derive shared key $k_{UE}\leftarrow KDF\left(epk_{UE}^{-1}\cdot pk_{HN}\right)$      Parse the shared key $k_{UE}$ as $\left(k_{1},k_{2}\right)$      Compute $C_{1}\leftarrow ENC\left(k_{1},SUPI\right)$ and $C_{2}\leftarrow MAC\left(k_{2},C_{1}\right)$      Set $SUCI\leftarrow (C_{0},C_{1},C_{2})$   *Outputs.* UE sends (SUCI) to SN.


**Algorithm 2** Initiation Step 1.2 [SN]
   *Inputs:* 
$SUCI,ID_{SN}$      SN add $ID_{SN}$ information and forward $\left(SUCI,ID_{SN}\right)$ to the HN   *Outputs.* SN sends $\left(SUCI,ID_{SN}\right)$ to HN.


**Algorithm 3** Initiation Step 1.3 [HN]
   *Inputs:* 
$\left(SUCI,ID_{SN}\right)$      Parse SUCI as $C_{0},C_{1},C_{2}$      Compute a shared key $k_{HN}\leftarrow KDF\left(sk_{HN}\cdot C_{0}\right)$      Parse the shared key $k_{HN}$ as $\left(k_{1},k_{2}\right)$      Compute $MAC^{\prime }\leftarrow MAC\left(k_{2},C_{1}\right)$      Check if $MAC^{\prime }=C_{2}$, Abort in the negative case. Otherwise, continue      Compute $SUPI\leftarrow DEC(k_{1},C_{1})$      Retrieve the corresponding *k* and $SQN_{HN}$ based on SUPI   *Outputs.* HN outputs ⊥ if $\;MAC^{\prime }\neq C_{2}$, Otherwise, it output $k,SQN_{HN}$.


#### 3.2.2. The Challenge–Response Phase: Step 2

During this phase, the UE and HN perform mutual authentication using a challenge–response mechanism and establish anchor keys (specifically, $K_{SEAF}$) with the SN. The HN initiates this process by generating an ephemeral public–private key pair $\left(epk_{HN},epk_{HN}^{-1}\right)$. Here, the ephemeral public key $epk_{HN}$ is designated as the random challenge RAND. The key DHK is derived through an ECDH key agreement using the ephemeral private key $epk_{HN}^{-1}$ in conjunction with the UE’s ephemeral public key $epk_{UE}$, provided from $C_{0}$. This DHK acts as a key material to ensure forward secrecy. This challenge–response process is shown in Algorithms 4–8.

**Algorithm 4** The Challenge–Response Step 2.1 [HN]
   *Inputs:* 
$k,SQN_{HN}$      Generate an ephemeral private-public key pair ($epk_{HN},epk_{HN}^{-1}$), where $epk_{HN}=epk_{HN}^{-1}\cdot G$      Compute a Diffie-Hellman key $DHK\leftarrow epk_{HN}^{-1}\cdot C_{0}$      Set $RAND\leftarrow epk_{HN}$ as a challenge      Compute $MAC\leftarrow f_{1}\left(k,SQN_{HN},AMF,RAND⊕DHK\right)$      Compute $AK\leftarrow f_{5}\left(k,RAND⊕DHK\right)$      Compute $CONC\leftarrow AK⊕SQN_{HN}$      Set AUTN←(CONC,AMF,MAC)      Compute $CK\leftarrow f_{3}\left(k,RAND⊕DHK\right)$ and $IK\leftarrow f_{4}\left(k,RAND⊕DHK\right)$      Compute expected response $XRES\leftarrow f_{2}\left(k,RAND⊕DHK\right)$      Compute $XRES^{*}\leftarrow KDF(CK∥IK,ID_{SN}∥RAND∥XRES)$      Compute $HXRES^{*}\leftarrow LEFT(128,H_{SHA-256}\left(RAND∥XRES^{*}\right))$      Derive $K_{AUSF}\leftarrow KDF(CK∥IK,ID_{SN}∥CONC∥DHK)$      Derive $K_{SEAF}\leftarrow KDF\left(K_{AUSF},ID_{SN}\right)$      Increase $SQN_{HN}\leftarrow SQN_{HN}+1$      Set $\mathrm{HE}-\mathrm{AV}\leftarrow (RAND,AUTN,XRES^{*},K_{AUSF})$      Set $\mathrm{SE}-\mathrm{AV}\leftarrow (RAND,AUTN,HXRES^{*})$   *Outputs.* HN sends (SE-AV) to SN.


**Algorithm 5** The Challenge–Response Step 2.2 [SN]
   *Inputs:* 
(SE-AV)      SN parse (SE-AV) as $\left(RAND,AUTN,HXRES^{*}\right)$      SN store $\left(RAND,HXRES^{*}\right)$ and sends (RAND,AUTN) to UE   *Outputs.* SN sends (RAND,AUTN) to UE.


**Algorithm 6** The Challenge–Response Step 2.3 [UE]
   *Inputs:* 
(RAND,AUTN)      UE computes a Diffie-Hellman key $DHK\leftarrow epk_{UE}^{-1}\cdot RAND$      Parse AUTN as (CONC,AMF,MAC)      Compute $AK\leftarrow f_{5}\left(k,RAND⊕DHK\right)$      De-conceal $SQN_{HN}\leftarrow AK⊕CONC$      Check if $f_{1}\left(k,SQN_{HN},AMF,RAND⊕DHK\right)$      
If this check does not pass, the UE returns ⊥ and sends a failure message Mac_Failure to SN (case i). Otherwise, proceed to the next step.      Check $SQN_{UE}<SQN_{HN}<SQN_{UE}+\Delta$
If this check does not pass, the SIM card computes$MAC^{*}\leftarrow f_{1}^{*}\left(k,SQN_{UE},AMF,RAND⊕DHK\right)$$AK^{*}\leftarrow f_{5}^{*}\left(k,RAND⊕DHK\right)$$AUTS\leftarrow (AK^{*}⊕SQN_{UE},AMF,MAC^{*})$After the SIM card computes AUTS, then UE re-synchronizes with HN by sending a failure message Sync_Failure and AUTS to SN (case ii)Otherwise, proceed to the next step.      Update $SQN_{UE}\leftarrow SQN_{HN}$      Compute $CK\leftarrow f_{3}\left(k,RAND⊕DHK\right)$ and $IK\leftarrow f_{4}\left(k,RAND⊕DHK\right)$      Compute $RES\leftarrow f_{2}\left(k,RAND⊕DHK\right)$      SIM return RES,CK and IK to ME      The ME compute $RES^{*}\leftarrow KDF(CK∥IK,ID_{SN}∥RAND∥RES)$      Derive $K_{AUSF}\leftarrow KDF(CK∥IK,ID_{SN}∥CONC∥DHK)$      Derive $K_{SEAF}\leftarrow KDF\left(K_{AUSF},ID_{SN}\right)$      The UE returns $\left(K_{SEAF},RES^{*}\right)$   *Outputs.* The UE stores $K_{SEAF}$ and sends $RES^{*}$ to SN (case iii)


**Algorithm 7** The Challenge–Response Step 2.4 [SN]
   *Inputs:* 
$RES^{*}$      SN compute $HRES^{*}\leftarrow LEFT\left(128,H_{SHA-256}\left(RAND∥RES^{*}\right)\right)$      Check if $HRES^{*}=HXRES^{*}$, Abort in the negative case. Otherwise, continue   *Outputs.* SN sends $RES^{*}$ to HN.


**Algorithm 8** The Challenge–Response Step 2.5 [HN]
   *Inputs:* 
$RES^{*}$      HN Check if $XRES^{*}=RES^{*}$, Abort in the negative case. Otherwise, continue   *Outputs.* HN sends $\left(Result,SUPI,K_{SEAF}\right)$ to SN.


## 4. Formal Verification

Our formal verification is conducted in accordance with the ISO/IEC 29128-1:2023 standard [12], which provides a rigorous framework for the formal verification of security protocols. Adhering to this standard ensures that our verification process is systematic and robust, enabling the effective identification of potential vulnerabilities within the 5G-AKA-FS protocol. Furthermore, this standard supports our objective of meeting stringent security requirements through the application of advanced formal methods.

To further enhance the accuracy and comprehensiveness of the security verification process, we adopted a hybrid approach combining SVO logic and the formal verification tool ProVerif. SVO logic builds upon BAN logic while incorporating insights from GNY [13], AT [14], and VO [15] logics, enabling precise identification of specific vulnerabilities. While BAN logic is relatively simple and foundational, SVO logic represents a more mature and specialized framework, capable of addressing complex security requirements more effectively. Notably, in this study, we partitioned the idealization process in SVO logic into three distinct stages, significantly reducing the likelihood of errors during verification. In contrast, BAN logic does not support such partitioning, resulting in a higher probability of errors. This structural enhancement in SVO logic substantially lowers error rates and improves its reliability.

Moreover, we extended SVO logic to incorporate perfect forward secrecy (PFS) properties, further reinforcing the rigor of our security analysis. This enhancement enhances the applicability of SVO logic to the 5G-AKA-FS protocol, establishing a foundation for more resilient and comprehensive security verification.

ProVerif was employed to verify the unbounded properties under an extended adversary model, enabling a layered security analysis. While ProVerif excels in evaluating general protocol properties, the integration of SVO logic complements ProVerif by addressing its limitations in precision, thereby ensuring both depth and breadth in the analysis. This dual-methodology approach facilitates a more thorough and robust verification of the 5G-AKA-FS protocol, effectively strengthening its security evaluation.

### 4.1. Correction of the BAN Logic Verification

This subsection focuses on addressing minor corrections in the BAN logic verification for the 5G-AKA-FS protocol as initially outlined in [3]. During our review, we identified four minor errors, including typographical mistakes and omissions of specific characters, which could compromise the clarity and precision of the formal security analysis. These corrections are essential to ensure the verification’s accuracy and to maintain the protocol’s integrity in its security guarantees. Each correction has been carefully validated to align with the original intent and logic structure, ensuring it is both necessary and consistent with formal verification principles.

1. On page 12, Formula (1), $UE\to HN:{\langle \{SUPI,UE\overset{K_{2}}{\Leftrightarrow }HN\}\rangle }_{k_{1}}$, contains an error. It should match the part seen by HN in Formula (7) on page 12. This is needed because Formula (7) is derived from Formula (1). As a result, Formula (1) should be corrected to $UE\to HN:{\langle {\left\{SUPI,UE\overset{K_{2}}{\Leftrightarrow }HN\right\}}_{k_{2}},UE\overset{K_{1}}{\Leftrightarrow }HN\rangle }_{k_{1}}$.
2. On page 14, the Formula (40) $UE\;⊲{\langle SQN_{HN},AMF,\overset{Y}{\to }HN,UE\overset{K_{SEAF}}{↔}SEAF\rangle }_{k}$ by (11), is incorrect. It should include $UE\overset{x\cdot y\cdot G}{↔}HN$ and change $UE\overset{K_{SEAF}}{↔}SEAF$ to $UE\overset{K_{SEAF}}{↔}HN$ based on Formula (11). As a result, Formula (40) should be corrected to
  $UE⊲{\langle SQN_{HN},AMF,\overset{Y}{\to }HN,UE\overset{x\cdot y\cdot G}{↔}HN,UE\overset{K_{SEAF}}{↔}HN\rangle }_{k}$ by (11). Accordingly, Formulas (41) and (42) should be corrected as follows:
  - (41): $UE\;|\equiv \;HN\;|∼(SQN_{HN},AMF,\overset{Y}{\to }HN,UE\overset{x\cdot y\cdot G}{↔}HN,UE\overset{K_{SEAF}}{↔}\;HN)$ by (40), (15), MM.
  - (42): $UE\;|\equiv \;HN\;|\equiv (SQN_{HN},AMF,\overset{Y}{\to }HN,UE\overset{x\cdot y\cdot G}{↔}HN,UE\overset{K_{SEAF}}{↔}HN)$ by (41), (18), FR, NV.
3. On page 15, Formula (49), $SN\;|\equiv \;RES^{*}$ by (48), (18), (19), HR, is incorrect because it is derived from Formula (18), which does not align with the derivation required for the equivalence. This formula should be corrected to $SN\;|\equiv \;RES^{*}$ by (48), (19), (20), HR. This adjustment is crucial because Formula (20) appropriately justifies the derivation.
4. On page 15, Formula (54), $SN\;⊲\;{\left\{SUPI,UE\overset{K_{SEAF}}{↔}HN,(UE\overset{K_{SEAF}}{↔}SEAF)\right\}}_{K_{N12}}$ by (14) contains a typographical error. The reference to SEAF instead of HN misrepresents the intended derivation. Furthermore, Formula (54) omits the belief $UE\overset{K_{SEAF}}{↔}HN$ to UE, which is essential for advancing the derivation. This formula should be revised to
  $$SN\;⊲\;{\left\{SUPI,UE\overset{K_{SEAF}}{↔}HN,(UE\;|\equiv \;UE\overset{K_{SEAF}}{↔}HN)\right\}}_{K_{N12}}\;\mathrm{by}\;(14)$$

### 4.2. Extension of SVO Logic

This section provides a brief overview of SVO logic and its extension for the formal verification of the 5G-AKA-FS protocol.

#### 4.2.1. SVO Logic

The notation and axioms of SVO logic used in this paper are shown in Table 3 and Table 4.

#### 4.2.2. Extension

No formal axiom addresses PFS in SVO logic. To address this gap, we introduce new notation and a corresponding axiom specifically for PFS. This addition formalizes the conditions under which PFS is preserved, providing a framework for analyzing protocols that require this security property.

##### **New** **Notation**

We start by introducing the new notation for the axiom, as displayed in Table 5.

##### **New** **Axiom**

We present the new axiom for the 5G-AKA-FS protocol, named the perfect forward secrecy axiom (PFSA). For this axiom to hold, two assumptions must be defined.

**Assumption** **1.** *A set of ephemeral public keys $eph(\overset{‿}{\mathrm{PK}})$ is a union of the subset of ephemeral public keys from the set $eph(\overset{‿}{\mathrm{PK}})$ owned by P and the subset of ephemeral public keys from the set $eph(\overset{‿}{\mathrm{PK}})$ owned by Q, denoted as*

$$eph(\overset{‿}{\mathrm{PK}})=own(P,eph(\overset{‿}{\mathrm{PK}}))\cup own(Q,eph(\overset{‿}{\mathrm{PK}}))$$

**Assumption** **2.** *The subset of ephemeral public keys from the set $eph(\overset{‿}{\mathrm{PK}})$ owned by P and the subset of ephemeral public keys from the set $eph(\overset{‿}{\mathrm{PK}})$ owned by Q are mutually exclusive, and denoted as*

$$own(P,eph(\overset{‿}{\mathrm{PK}}))\;\cap \;own(Q,eph(\overset{‿}{\mathrm{PK}}))=\emptyset$$

Once the two assumptions are satisfied, we can proceed to define the perfect forward secrecy axiom.

**Axiom** **1** (Perfect forward secrecy)**.** *The perfect forward secrecy axiom (PFSA) is designed to account for two cases. For a set of public keys $\overset{‿}{\mathrm{PK}}$, if $|\overset{‿}{\mathrm{PK}}|>1$ then,*

(1) $$own(P,eph\left(\overset{‿}{\mathrm{PK}}\right))\wedge \;Q\;says\;own(Q,eph\left(\overset{‿}{\mathrm{PK}}\right))\to P\;\mathrm{allows}\;\mathrm{fps}(P\overset{F_{0}\left(\overset{‿}{\mathrm{PK}}\right)}{↔}Q)$$

(2) $$own(Q,eph\left(\overset{‿}{\mathrm{PK}}\right))\wedge \;P\;says\;own(P,eph\left(\overset{‿}{\mathrm{PK}}\right))\to Q\;\mathrm{allows}\;\mathrm{fps}(P\overset{F_{0}\left(\overset{‿}{\mathrm{PK}}\right)}{↔}Q)$$

*if conditions (1) and (2) hold, then the key $P\overset{F_{0}\left(\overset{‿}{\mathrm{PK}}\right)}{↔}Q$ satisfies perfect forward secrecy.*

*On the other hand, if the set $\overset{‿}{\mathrm{PK}}$ contains only one element pk, then*

(3) $$own(P,eph\left(pk\right))\wedge \;Q\;says\;\{P\overset{K}{↔}Q{\}}_{pk}\to P\;\mathrm{allows}\;\mathrm{fps}(P\overset{K}{↔}Q)$$

*If condition (3) holds, then the key $P\overset{F_{0}\left(\overset{‿}{\mathrm{PK}}\right)}{↔}Q$ satisfies perfect forward secrecy. Note that in this case, the public key pk is a ciphering public key.*

### 4.3. Formal Verification of 5G-AKA-FS Using Enhanced SVO Logic

In this section, we perform a comprehensive security analysis of the 5G-AKA-FS protocol through formal verification, leveraging an enhanced SVO logic. This approach is augmented by including a novel perfect forward secrecy axiom formulated to examine and validate long-term security properties within the protocol rigorously.

The protocol analysis of SVO logic starts with defining initial state assumptions and detailing the protocol’s starting conditions without idealization, unlike in BAN logic. This is followed by protocol annotation, where messages are represented directly from the specification, including plaintext elements. Next, comprehension assumptions outline what each principal understands upon receiving messages, while interpretation assumptions clarify how these messages are understood based on protocol design, focusing on the receiver’s perspective rather than just the sender’s intent. Finally, SVO logic derives the beliefs of protocol principals through structured rules and reasoning. If these derivations succeed, the protocol is marked secure; otherwise, it is considered insecure, prompting iterative adjustments until security is achieved.

#### 4.3.1. Initial State Assumptions

As the first step, we define the initial state assumptions regarding the 5G-AKA-FS protocol.

(4) $$HN\;believes\;PK_{\delta }\left(HN,pk_{HN}\right)$$

(5) $$HN\;believes\;PK_{\delta }\left(HN,epk_{HN}\right)$$

(6) $$HN\;believes\;PK_{\delta }\left(UE,epk_{UE}\right)$$

(7) $$HN\;believes\;\mathrm{fresh}(epk_{UE})$$

(8) $$SN\;believes\;SN\overset{K_{sh}}{↔}HN$$

(9) SNbelievesfresh(n)

(10) $$SN\;believes\;HN\;controls\;UE\overset{HXRES^{*}}{↔}SN$$

(11) $$SN\;believes\;HN\;controls\;fresh(HXRES^{*})$$

(12) $$UE\;believes\;UE\overset{K}{↔}HN$$

(13) UEbelievesfresh(SQN)

(14) $$UE\;believes\;HN\;controls\;PK_{\delta }\left(HN,epk_{HN}\right)$$

(15) $$UE\;believes\;PK_{\delta }\left(UE,epk_{UE}\right)$$

(16) $$UE\;believes\;eph(epk_{UE})$$

(17) $$HN\;believes\;UE\overset{K}{↔}HN$$

(18) $$HN\;believes\;fresh(epk_{HN})$$

(19) $$HN\;believes\;UE\;controls\;PK_{\delta }\left(UE,epk_{UE}\right)$$

(20) $$HN\;believes\;UE\;controls\;fresh(epk_{UE})$$

(21) $$HN\;believes\;eph(epk_{HN})$$

(22) SNbelievesfresh(m)

(23) $$SN\;believes\;HN\;controls\;UE\overset{K_{SEAF}}{↔}HN$$

#### 4.3.2. Received Message Assumptions

With the initial assumptions established, the 5G-AKA-FS protocol is subsequently annotated as follows.

(24) $$HN\;received\;(epk_{UE},{\left\{SUPI\right\}}_{K_{1}},\{{\left\{SUPI\right\}}_{k_{1}}{\}}_{k_{2}},ID_{SN})$$

(25) $$\begin{array}{c} SN\;received\;\{epk_{HN},\left({\left\{SQN\right\}}_{AK},AMF,{\left\{SQN,AMF,epk_{HN},DHK\right\}}_{K}\right),\\ \;HXRES^{*}{,n\}}_{K_{sh}} \end{array}$$

(26) $$UE\;received\;(epk_{HN},\left({\left\{SQN\right\}}_{AK},AMF,{\left\{SQN,AMF,epk_{HN},DHK\right\}}_{K}\right))$$

(27) $$SN\;received\;RES^{*}$$

(28) $$HN\;received\;RES^{*}$$

(29) $$SN\;received\;{\left\{SUPI,K_{SEAF},m\right\}}_{K_{sh}}$$

#### 4.3.3. Comprehension Assumptions (Necessitation Application)

In this step, we outline how each principal comprehends the received message, as detailed below:

(30) $$HN\;believes\;HN\;received\;({\langle epk_{UE}\rangle }_{*HN},{\left\{SUPI\right\}}_{k_{1}},\{{\left\{SUPI\right\}}_{k_{1}}{\}}_{k_{2}},ID_{SN})$$

(31) $$\begin{array}{c} SN\;believes\;SN\;received\;\{{\langle epk_{HN}\rangle }_{*SN},({\langle CONC\rangle }_{*SN},AMF,{\langle MAC\rangle }_{*SN}),\\ \;{\langle HXRES^{*}\rangle }_{*SN},n{\}}_{K_{sh}} \end{array}$$

(32) $$\begin{array}{c} UE\;believes\;UE\;received\;({\langle epk_{HN}\rangle }_{*UE},({\left\{SQN\right\}}_{{\langle AK\rangle }_{*UE}},AMF,\\ \;{\left\{SQN,AMF,{\langle epk_{HN}\rangle }_{*UE},{\langle DHK\rangle }_{*UE}\right\}}_{K})) \end{array}$$

(33) $$SN\;believes\;SN\;received\;{\langle {RES}^{*}\rangle }_{*SN}$$

(34) $$HN\;believes\;HN\;received\;{\left\{ID_{SN},epk_{HN},{\left\{epk_{HN},DHK\right\}}_{K}\;\mathrm{from}\mathrm{UE}\right\}}_{ICK}$$

(35) $$SN\;believes\;SN\;received\;{\left\{\{{\langle SUPI\rangle }_{*SN},{\langle K_{SEAF}\rangle }_{*SN},m\}\right\}}_{K_{sh}}$$

where

$$\begin{array}{cc} CONC & ={\left\{SQN\right\}}_{AK};\\ MAC & ={\left\{SQN,AMF,epk_{HN},DHK\right\}}_{K};\\ RES^{*} & ={\left\{ID_{SN},epk_{HN},{\left\{epk_{HN},DHK\right\}}_{K}\;\mathrm{from}\mathrm{UE}\right\}}_{ICK}. \end{array}$$

#### 4.3.4. Interpretation Assumptions

In this step, we illustrate how each principal interprets the comprehended message, as described below

(36) $$\begin{array}{c} HN\;believes\;HN\;received\;({\langle epk_{UE}\rangle }_{*HN},{\left\{SUPI\right\}}_{k_{1}},\{{\left\{SUPI\right\}}_{k_{1}}{\}}_{k_{2}},ID_{SN})\;\to \\ \;HN\;believes\;HN\;received\;\left(\begin{array}{c} PK_{\delta }\left(UE,{\langle epk_{UE}\rangle }_{*HN}\right),\\ {\left\{\begin{array}{c} {\left\{SUPI,PK_{\delta }\left(UE,{\langle {epk}_{UE}\rangle }_{*HN}\right)\right\}}_{k_{1}},\\ PK_{\delta }\left(UE,{\langle {epk}_{UE}\rangle }_{*HN}\right) \end{array}\right\}}_{k_{2}} \end{array}\right) \end{array}$$

(37) $$\begin{array}{c} SN\;believes\;SN\;received\;{\left\{\begin{array}{c} {\langle epk_{HN}\rangle }_{*SN},({\langle CONC\rangle }_{*SN},AMF,{\langle MAC\rangle }_{*SN}),\\ {\langle HXRES^{*}\rangle }_{*SN},n \end{array}\right\}}_{K_{sh}}\to \\ \;SN\;believes\;SN\;received\;{\left\{\begin{array}{c} PK_{\delta }\left(HN,{\langle epk_{HN}\rangle }_{*SN}\right),\\ ({\langle CONC\rangle }_{*SN},AMF,{\langle MAC\rangle }_{*SN}),\\ UE\overset{{\langle HXRES^{*}\rangle }_{*SN}}{↔}SN,\mathrm{fresh}\left({\langle HXRES^{*}\rangle }_{*SN}\right),n \end{array}\right\}}_{K_{sh}} \end{array}$$

(38) $$\begin{array}{c} UE\;believes\;UE\;received\;\left(\begin{array}{c} {\langle {epk}_{HN}\rangle }_{*UE},({\left\{SQN\right\}}_{{\langle AK\rangle }_{*UE}},AMF,\\ {\left\{SQN,AMF,{\langle {epk}_{HN}\rangle }_{*UE},{\langle DHK\rangle }_{*UE}\right\}}_{K}) \end{array}\right)\;\to \\ \;{UE\;believes\;UE\;received\;\left\{\begin{array}{c} {SQN,AMF,PK}_{\delta }\left(HN,{\langle {epk}_{HN}\rangle }_{*UE}\right),\\ eph\left({\langle {epk}_{HN}\rangle }_{*UE}\right),UE\overset{{\langle DHK\rangle }_{*UE}}{↔}HN \end{array}\right\}}_{K} \end{array}$$

(39) $$\begin{array}{c} (SN\;believes\;SN\;received\;{\langle {RES}^{*}\rangle }_{*SN})\;\wedge \;(SN\;believes\;UE\overset{{\langle HXRES^{*}\rangle }_{*SN}}{↔}SN)\;\to \\ \;SN\;believes\;SN\;received{\left\{{\langle RES^{*}\rangle }_{*SN},{\langle HXRES^{*}\rangle }_{*SN}\right\}}_{{\langle HXRES^{*}\rangle }_{*SN}} \end{array}$$

(40) $$\begin{array}{c} HN\;believes\;HN\;received\;{\left\{ID_{SN},epk_{HN},{\left\{epk_{HN},DHK\right\}}_{K}\;\mathrm{from}\mathrm{UE}\right\}}_{ICK}\;\;\wedge \\ \;(HN\;believes\;UE\overset{ICK}{↔}HN)\;\to \\ \;HN\;believes\;HN\;received\;{\left\{\begin{array}{c} ID_{SN},PK_{\delta }\left(HN,epk_{HN}\right),\\ {\left\{\begin{array}{c} PK_{\delta }\left(HN,epk_{HN}\right),PK_{\delta }\left(UE,{\langle epk_{UE}\rangle }_{*HN}\right),\\ \mathrm{fresh}\left({\langle epk_{UE}\rangle }_{*HN}\right),eph\left({\langle epk_{UE}\rangle }_{*HN}\right),\\ UE\overset{{\langle DHK\rangle }_{*HN}}{↔}HN \end{array}\right\}}_{K} \end{array}\right\}}_{ICK} \end{array}$$

(41) $$\begin{array}{c} SN\;believes\;SN\;received\;{\left\{\{{\langle SUPI\rangle }_{*SN},{\langle K_{SEAF}\rangle }_{*SN},m\}\right\}}_{K_{sh}}\to \\ \;SN\;believes\;SN\;received\;{\left\{{\langle SUPI\rangle }_{*SN},UE\overset{{\langle K_{SEAF}\rangle }_{*SN}}{↔}SN,m\right\}}_{K_{sh}} \end{array}$$

where

$$\begin{array}{cc} eph(epk_{HN}) & =own(HN,eph(\left\{epk_{HN},epk_{UE}\right\}));\\ eph(epk_{UE}) & =own(UE,eph(\left\{epk_{HN},epk_{UE}\right\})). \end{array}$$

#### 4.3.5. Derivation

In the final step, we iteratively apply axioms, inference rules, and the newly incorporated forward perfect secrecy axiom until the desired results are achieved.

From (36), we derive

(42) $$HN\;believes\;HN\;received\;(PK_{\delta }\left(UE,{\langle epk_{UE}\rangle }_{*HN}\right),{\left\{\begin{array}{c} {\left\{SUPI,PK_{\delta }\left(UE,{\langle {epk}_{UE}\rangle }_{*HN}\right)\right\}}_{k_{1}},\\ PK_{\delta }\left(UE,{\langle {epk}_{UE}\rangle }_{*HN}\right) \end{array}\right\}}_{k_{2}})$$

by (30), MP,

(43) $$HN\;believes\;HN\;received\;PK_{\delta }\left(UE,{\langle epk_{UE}\rangle }_{*HN}\right)$$

by (42), RA, BA,

(44) $$HN\;believes\;HN\;has\;PK_{\delta }\left(UE,{\langle epk_{UE}\rangle }_{*HN}\right)$$

by (43), PA, BA,

(45) $$HN\;believes\;UE\overset{{pk}_{HN}^{-1}\cdot {\langle epk_{UE}\rangle }_{*HN}}{↔}HN$$

by (4), (6), KA, BA,

(46) $$HN\;believes\;UE\overset{k_{1}}{↔}HN$$

by (45),

(47) $$HN\;believes\;UE\overset{k_{2}}{↔}HN$$

by (45),

(48) $$HN\;believes\;UE\;said\;({\left\{SUPI,PK_{\delta }\left(UE,{\langle {epk}_{UE}\rangle }_{*HN}\right)\right\}}_{k_{1}},PK_{\delta }\left(UE,{\langle {epk}_{UE}\rangle }_{*HN}\right))$$

by (42), RA, (47), SAA, BA,

(49) $$HN\;believes\;UE\;says\;({\left\{SUPI,PK_{\delta }\left(UE,{\langle {epk}_{UE}\rangle }_{*HN}\right)\right\}}_{k_{1}},PK_{\delta }\left(UE,{\langle {epk}_{UE}\rangle }_{*HN}\right))$$

by (48), (7), FR, NV, BA,

(50) $$HN\;believes\;HN\;received\;{\left\{SUPI,PK_{\delta }\left(UE,{\langle {epk}_{UE}\rangle }_{*HN}\right)\right\}}_{k_{1}}$$

by (48), SA, BA,

(51a) $$HN\;believes\;UE\;says\;(SUPI,PK_{\delta }\left(UE,{\langle {epk}_{UE}\rangle }_{*HN}\right))$$

by (50), (46), SAA, (7), FR, NV, BA,

(51b) HNbelievesUEsaysSUPI

by (51a), SA, BA,

(51c) $$HN\;believes\;UE\overset{DHK}{↔}HN$$

by (5), (6), KA, BA,

where

$$\begin{array}{cc} DHK & =epk_{HN}^{-1}\cdot {\langle epk_{UE}\rangle }_{*HN} \end{array}$$

Derivations (44) and (51b) reach a point where they cannot be extended further and thus must terminate at this stage. To enable continued analysis, hypothesis (6) is introduced as an additional assumption. Similarly, derivations (48) and (50) also reach their limits and cannot proceed further, requiring the addition of hypothesis (7) to support further analysis.

From (37), we derive

(52) $$SN\;believes\;SN\;received\;{\left\{\begin{array}{c} PK_{\delta }\left(HN,{\langle epk_{HN}\rangle }_{*SN}\right),\left({\langle CONC\rangle }_{*SN},AMF,{\langle MAC\rangle }_{*SN}\right),\\ UE\overset{{\langle HXRES^{*}\rangle }_{*SN}}{↔}SN,\mathrm{fresh}\left({\langle HXRES^{*}\rangle }_{*SN}\right),n \end{array}\right\}}_{K_{sh}}$$

by (31), (37), MP,

(53) $$SN\;believes\;HN\;said\left(\begin{array}{c} PK_{\delta }\left(HN,{\langle epk_{HN}\rangle }_{*SN}\right),\left({\langle CONC\rangle }_{*SN},AMF,{\langle MAC\rangle }_{*SN}\right),\\ UE\overset{{\langle HXRES^{*}\rangle }_{*SN}}{↔}SN,\mathrm{fresh}\left({\langle HXRES^{*}\rangle }_{*SN}\right),n \end{array}\right)$$

by (52), (8), SAA, BA,

(54) $$SN\;believes\;HN\;says\left(\begin{array}{c} PK_{\delta }\left(HN,{\langle epk_{HN}\rangle }_{*SN}\right),\left({\langle CONC\rangle }_{*SN},AMF,{\langle MAC\rangle }_{*SN}\right),\\ UE\overset{{\langle HXRES^{*}\rangle }_{*SN}}{↔}SN,\mathrm{fresh}\left({\langle HXRES^{*}\rangle }_{*SN}\right),n \end{array}\right)$$

by (53), (9), FR, NV, BA,

(55) $$SN\;believes\;HN\;says\;UE\overset{{\langle HXRES^{*}\rangle }_{*SN}}{↔}SN$$

by (54), SA, BA,

(56) $$SN\;believes\;UE\overset{{\langle HXRES^{*}\rangle }_{*SN}}{↔}SN$$

by (55), (10), JR, BA,

(57) $$SN\;believes\;\mathrm{fresh}({\langle HXRES^{*}\rangle }_{*SN})$$

by (54), SA, (11), JR, BA.

From (38), we derive

(58) $$UE\;believes\;UE\;received\;{\left\{\begin{array}{c} SQN,AMF,PK_{\delta }\left(HN,{\langle {epk}_{HN}\rangle }_{*UE}\right),\\ eph\left({\langle {epk}_{HN}\rangle }_{*UE}\right),UE\overset{{\langle DHK\rangle }_{*UE}}{↔}HN \end{array}\right\}}_{K}$$

by (32), (38), MP,

(59) $$UE\;believes\;UE\;said\;\left(\begin{array}{c} SQN,AMF,PK_{\delta }\left(HN,{\langle {epk}_{HN}\rangle }_{*UE}\right),eph\left({\langle {epk}_{HN}\rangle }_{*UE}\right),\\ UE\overset{{\langle DHK\rangle }_{*UE}}{↔}HN \end{array}\right)$$

by (58), (12), SAA, BA,

(60) $$UE\;believes\;HN\;says\;\left(\begin{array}{c} SQN,AMF,PK_{\delta }\left(HN,{\langle {epk}_{HN}\rangle }_{*UE}\right),eph\left({\langle {epk}_{HN}\rangle }_{*UE}\right),\\ UE\overset{{\langle DHK\rangle }_{*UE}}{↔}HN \end{array}\right)$$

by (59), (13), FR, NV, BA,

(61) $$UE\;believes\;PK_{\delta }\left(HN,{\langle {epk}_{HN}\rangle }_{*UE}\right)$$

by (60), SA, (14), JR, BA,

(62) $$UE\;believes\;UE\overset{{epk}_{UE}^{-1}\cdot {\langle epk_{HN}\rangle }_{*UE}\cdot G}{↔}HN$$

by (15), (61), KA, BA,

(63) $$UE\;believes\;UE\overset{{\langle DHK\rangle }_{*UE}}{↔}HN$$

by (62),

(64) $$UE\;believes\;HN\;says\;UE\overset{{\langle DHK\rangle }_{*UE}}{↔}HN$$

by (60), SA, BA,

(65) $$UE\;believes\;HN\;says\;eph({\langle {epk}_{HN}\rangle }_{*UE})$$

by (60), SA, BA,

(66) $$UE\;\mathrm{allows}\;\mathrm{fps}(UE\overset{{\langle DHK\rangle }_{*UE}}{↔}HN)$$

by (16), (65), PFSA, BA,

(67) $$UE\;\mathrm{believes}\;UE\overset{K_{SEAF}}{↔}HN$$

by (12), (60), SA, (63), BA.

From (39), we derive

(68) $$SN\;believes\;SN\;received{\left\{{\langle RES^{*}\rangle }_{*SN},{\langle HXRES^{*}\rangle }_{*SN}\right\}}_{{\langle HXRES^{*}\rangle }_{*SN}}$$

by (33), (56), (39), MP,

(69) $$SN\;believes\;UE\;said({\langle RES^{*}\rangle }_{*SN},{\langle HXRES^{*}\rangle }_{*SN})$$

by (68), (56), SAA, BA,

(70) $$SN\;believes\;UE\;says({\langle RES^{*}\rangle }_{*SN},{\langle HXRES^{*}\rangle }_{*SN})$$

by (69), (57), FR, NV, BA,

(71) $$SN\;believes\;UE\;says{\langle RES^{*}\rangle }_{*SN}$$

by (70), SA, BA.

From (40), we derive

(72) $$HN\;believes\;UE\overset{ICK}{↔}HN$$

by (5), (51c), (17)

(73) $$\begin{array}{c} HN\;believes\;HN\;received\;{\left\{ID_{SN},PK_{\delta }\left(HN,epk_{HN}\right),{\left\{\begin{array}{c} PK_{\delta }\left(HN,epk_{HN}\right),\\ PK_{\delta }\left(UE,{\langle epk_{UE}\rangle }_{*HN}\right),\\ \mathrm{fresh}({\langle epk_{UE}\rangle }_{*HN}),\\ eph({\langle epk_{UE}\rangle }_{*HN}),\\ UE\overset{{\langle DHK\rangle }_{*HN}}{↔}HN \end{array}\right\}}_{K}\right\}}_{ICK} \end{array}$$

by (34), (72), (40), MP,

(74) $$\begin{array}{c} HN\;believes\;UE\;says\;\left(ID_{SN},PK_{\delta }\left(HN,epk_{HN}\right),{\left\{\begin{array}{c} PK_{\delta }\left(HN,epk_{HN}\right),\\ PK_{\delta }\left(UE,{\langle epk_{UE}\rangle }_{*HN}\right),\\ \mathrm{fresh}({\langle epk_{UE}\rangle }_{*HN}),\\ eph({\langle epk_{UE}\rangle }_{*HN}),\\ UE\overset{{\langle DHK\rangle }_{*HN}}{↔}HN \end{array}\right\}}_{K}\right) \end{array}$$

by (72), (73), SAA, (18), FR, NV, BA,

(75) $$\begin{array}{c} HN\;believes\;UE\;says\;\left(\begin{array}{c} PK_{\delta }\left(HN,epk_{HN}\right),PK_{\delta }\left(UE,{\langle epk_{UE}\rangle }_{*HN}\right),\\ \mathrm{fresh}\left({\langle epk_{UE}\rangle }_{*HN}\right),eph\left({\langle epk_{UE}\rangle }_{*HN}\right),\\ UE\overset{{\langle DHK\rangle }_{*HN}}{↔}HN \end{array}\right) \end{array}$$

by (74), SA, (72), SAA, (18), FR, NV, BA,

(76) $$HN\;believes\;PK_{\delta }\left(UE,{\langle epk_{UE}\rangle }_{*HN}\right)$$

by (75), SA, (19), JR, BA,

(77) $$HN\;believes\;\mathrm{fresh}({\langle epk_{UE}\rangle }_{*HN})$$

by (75), SA, (20), JR, BA,

(78) $$HN\;believes\;UE\;says\;eph({\langle epk_{UE}\rangle }_{*HN})$$

by (75), SA, BA,

(79) $$HN\;allows\;\mathrm{fps}(UE\overset{{\langle DHK\rangle }_{*HN}}{↔}HN)$$

by (21), (75), PFSA, BA,

(80) $$HN\;believes\;UE\;says\;UE\overset{{\langle DHK\rangle }_{*HN}}{↔}HN$$

by (75), SA, BA,

where

$$\begin{array}{cc} eph(epk_{HN}) & =own(HN,eph(\left\{epk_{HN},epk_{UE}\right\}));\\ eph(epk_{UE}) & =own(UE,eph(\left\{epk_{HN},epk_{UE}\right\})). \end{array}$$

From (41), we derive

(81) $$SN\;believes\;SN\;received\;{\left\{{\langle SUPI\rangle }_{*SN},UE\overset{{\langle K_{SEAF}\rangle }_{*SN}}{↔}SN,m\right\}}_{K_{sh}}$$

by (35), (41), MP,

(82) $$SN\;believes\;HN\;said\;({\langle SUPI\rangle }_{*SN},UE\overset{{\langle K_{SEAF}\rangle }_{*SN}}{↔}SN,m)$$

by (81), (8), SAA, BA,

(83) $$SN\;believes\;HN\;says\;({\langle SUPI\rangle }_{*SN},UE\overset{{\langle K_{SEAF}\rangle }_{*SN}}{↔}SN,m)$$

by (82), (22), FR, NV, BA,

(84) $$SN\;believes\;HN\;says\;UE\overset{{\langle K_{SEAF}\rangle }_{*SN}}{↔}SN$$

by (83), SA, BA,

(85) $$SN\;believes\;UE\overset{{\langle K_{SEAF}\rangle }_{*SN}}{↔}SN$$

by (84), (23), JR, BA.

#### 4.3.6. Results

We derive the following lemmas from the formal analysis above, demonstrating that the security requirements are met.

**Lemma** **1.** *The 5G-AKA-FS protocol provides a secure key exchange.*

**Proof.**  Firstly, derivations (63) and (64) confirm the UE has successfully established the trust of DHK. Conversely, derivations (76), (51c), and (80) indicate the HN has successfully obtained the trust of both the DHK and UE’s ephemeral public key. The key agreement of DHK is shown by indirect trust from the derivations (64) and (80), while direct trust in DHK’s key is provided by derivations (63) and (76). Secondly, the belief (67) shows that the UE has obtained the trust in $K_{SEAF}$, while the belief (85) shows the SN has similarly obtained trust in $K_{SEAF}$. This proves that a secure key exchange has been successfully achieved between the UE and 5G core networks (SN and HN).    □

**Lemma** **2.** *The 5G-AKA-FS protocol provides mutual authentication between the UE and HN.*

**Proof.**  Derivation (64) confirms the authentication of the HN to the UE. Similarly, derivation (80) establishes the authentication of the UE to the HN. Moreover, derivation (71) shows the authentication of the SN to the UE. Consequently, this proves that mutual authentication between the UE and the 5G core networks (SN and HN) is achieved.    □

**Lemma** **3.** *The 5G-AKA-FS protocol provides perfect forward secrecy.*

**Proof.**  Based on derivations (66) and (79) and supported by the novel perfect forward secrecy axiom, it is proved that the key $UE\overset{{\langle DHK\rangle }_{*HN}}{↔}HN$ achieves perfect forward secrecy.    □

**Lemma** **4.** *The 5G-AKA-FS protocol provides resistance against active attack by a malicious SN.*

**Proof.**  The SN’s initial belief (71) leads to the HN’s belief (80), which in turn allows the SN to derive the beliefs (84) and (85), related to the anchor key $K_{SEAF}$. This sequence ensures that without (71) and (80), the SN cannot derive (84) and (85), thus preventing the SN from obtaining unauthorized access to the anchor key $K_{SEAF}$. This dependency blocks a malicious SN from preemptively accessing the anchor key $K_{SEAF}$, proving that the 5G-AKA-FS protocol resists attacks by malicious SNs.    □

**Lemma** **5.** *The 5G-AKA-FS protocol provides SUPI concealment.*

**Proof.**  First, hypothesis (6) assumes that the HN trusts an ephemeral public key $epk_{UE}$, and hypothesis (7) assumes that the HN trusts the freshness of $epk_{UE}$. Then, derivations (76) and (77) prove hypotheses (6) and (7), respectively. This implies that derivation (51b) is held, stating that the HN trust UE according to SUPI. Proving that the 5G-AKA-FS protocol provides SUPI concealment.    □

The results of our formal verification demonstrate the importance of introducing new axioms to support perfect forward secrecy (PFS) within the SVO logic framework. Unlike BAN logic, which does not natively address PFS, the extended SVO logic explicitly analyzes and validates this critical security property, enhancing the protocol’s reliability. Furthermore, SVO logic provides a stronger and more comprehensive verification framework compared to BAN logic, with its ability to rigorously evaluate advanced security properties.

### 4.4. Enhanced Formal Verification by ProVerif

The ProVerif model comprises three main stages: declaration, process macro, and the main process. Initially, as shown in Algorithm 9, the essential components for protocol analysis—such as types, constants, variables, channels, and security verification queries—are declared. Following this, the entities UE, SN, and HN are modularly structured as process macros, as presented in Algorithms 10, 11 and 12, respectively, allowing for systematic and reusable modeling of these entities.

We implement the processes under the Dolev–Yao attacker model [16], wherein the attacker is permitted access to encrypted data exclusively if they possess the requisite decryption key. Specifically, this process is developed in two scenarios: one where a verification scenario is established within a general authentication environment, as presented in Algorithm 13; and another where the long-term key exposure to an adversary with enhanced capabilities is assumed, as shown in Algorithm 14, allowing for a more rigorous evaluation at the unbounded level. To strengthen the evaluation of perfect forward secrecy, ProVerif supports the use of a dedicated function called phase. This function enables the simulation of an attacker with prior knowledge of secrets before the protocol execution, effectively enhancing the attacker model. By incorporating this feature, the protocol’s ability to maintain security despite advanced adversarial conditions is rigorously verified, ensuring robust protection against potential breaches.

#### 4.4.1. Query Analysis

Our verification targets key security requirements, such as mutual authentication, secure key exchange, SUPI concealment, and PFS. These security requirements are summarized from [3,6,7,8,9]. For each requirement, we define targeted queries, as detailed in Table 6, to thoroughly assess the protocol’s security properties.

The ‘inj-event’ function applies across each communication stage to ensure robust verification, thereby confirming both authentication and message freshness. For mutual authentication, the queries Q3 and Q4 are defined and assessed to evaluate the freshness of messages exchanged and authentication. Also, they verify whether the SN and HN sequentially authenticate the UE by validating the RES* value and then check if the SN securely receives the SUPI and anchor key $K_{SEAF}$. This allows for verification of whether a malicious SN could gain premature access to the anchor key without completing the final authentication of the UE. The security of the key exchange process is verified through the query Q1, ensuring that the anchor key $K_{SEAF}$ is securely negotiated without unauthorized access by an adversary. Additionally, the query Q0 is used to verify whether the confidentiality of the SUPI is ensured, while Q2 is employed to assess if the SUCI meets freshness requirements and to determine whether an attacker can reuse it to conduct resource exhaustion attacks. Lastly, the phase function in the main process is employed to confirm the preservation of PFS. In this case, ProVerif intentionally discloses secrets $sk_{HN}$ and *K* to the public in phase 1.

**Algorithm 9** Declaration and Queries       (* Channel specification *)       free sch: channel [private].       free usch: channel.              (* 5G functions *)       fun f1, f2, f3, f4, f5.              (* Secrecy verification *)       **Q0:** query attacker (SUPI).       **Q1:** query attacker (kseaf).              (* Security requirements verification *)       **Q2:** supi: bitstring, uepk: pubKey, hnpk: pubKey;               inj-event(endUE_HN_SUPI(supi, uepk, hnpk))               ==> inj-event(beginUE_HN_SUPI(supi, uepk, hnpk)).       **Q3:** supi: bitstring, *K*: key, rand: pubKey, sqn: seq;               inj-event(endUE_HN_MAC(supi, *K*, rand, sqn))               ==> inj-event(beginHN_UE_MAC(supi, *K*, rand, sqn)).       **Q4:** supi: bitstring, *K*: key, rand: pubKey, sqn: seq, res’: bitstring, kseaf: key;               inj-event(endSN_ANCHOR_KEY(supi, Kseaf))               ==> (inj-event(middleHN_RES(supi, *K*, rand, sqn))               ==> (inj-event(middleSN_RES(res’, rand))               ==> inj-event(beginUE_RES(supi, *K*, rand, sqn))

**Algorithm 10** proc_UE
   **Played by:** UE   **Input:** SUPI, sqn, pkHN, *K*      **[Step 1.1]**      new skUE;      let (c0, c1, c2) = calc_ue_suci(skUE, SUPI, pkHN) in      **event beginUE_HN_SUPI(SUPI, c0, pkHN);**      out(usch, (c0, c1, c2));   **[Step 2.3]**      **in**(usch, (RAND, CONC, AMF, MAC1));      let DHK = DHkey(sk(c0), RAND) in      let (MAC2, SQN, AK) = calc_ue_mac(RAND, DHK, *K*, CONC, AMF) in      if MAC1 <> MAC2 true then MACFailure;      if SQN <> calc_sqn(sqn, c0, RAND) true then SYNCFailure;      **event endUE_HN_MAC(SUPI, *K*, RAND, SQN);**      **event beginUE_RES(SUPI, *K*, RAND, SQN);**      let (RES’, HXRES’, KAUSF, KSEAF) = calc_hn_key(RAND, DHK, *K*, SQN, AK) in      **out**(usch, RES’);


**Algorithm 11** proc_SN
   **Played by:** SN   **Input:** SNname         **[Step 1.2]**      **in**(usch, (c0, c1, c2));      **out**(sch, (c0, c1, c2, SNname));   **[Step 2.2]**      **in**(sch, (RAND, CONC, AMF, MAC, HXRES’));      **out**(usch, (RAND, CONC, AMF, MAC));   **[Step 2.4]**      **in**(usch, RES’);      if HXRES’ <> SHA256((RES, RAND)) true then exit;      **event middleSN_RES(RES’, RAND);**      **out**(sch, RES’);   **[Step 2.6]**      **in**(sch, SUPI, KSEAF));      **event endSN_ANCHOR_KEY(SUPI, KSEAF);**


**Algorithm 12** proc_HN
   **Played by:** HN   **Input:** *K*, $sk_{HN}$, idHN         **[Step 1.3]**      **in**(sch,(c0, c1, c2, SNname));      new RAND: pubKey;      let (K1, K2) = get_hn_keys4supi(skHN, c0, c1, c2) in      if c2 <> hmac(K2, c1) && SUPI <> sdec(c1, K1) true then exit;      **event endUE_HN_SUPI(SUPI, c0, pk(skHN))**   **[Step 2.1]**      get ueDB(=SUPI, *K*, sqn) in      let DHK = DHkey(sk(RAND), c0) in      let (CONC, AK, MAC) = calc_hn_mac(RAND, DHK, *K*, SQN, AMF) in      let (XRES’, HXRES’, KAUSF, KSEAF) = calc_hn_key(RAND, DHK, *K*, SQN, AK) in      **event beginHN_UE_MAC(SUPI, *K*, RAND, SQN);**      out(sch, (RAND, CONC, AMF, MAC, HXRES’));   **[Step 2.5]**      **in**(sch, , RES’);      if XRES’ <> RES’ true then exit;      **event middleHN_RES(SUPI, *K*, RAND, SQN);**      **out**(sch, (SUPI, KSEAF));


**Algorithm 13** Main Process without FS
   process      new prHN: secKey; let puHN = pk(prHN) in out(usch, puHN);      new SUPI: bitstring; new *K*: key;      new SQN: seq;            insert ueDB(SUPI, *K*, SQN);      (!UE(SUPI, *K*, SQN, puHN))|(!SN(SNname))|(!HN(SUPI, prHN)))


**Algorithm 14** Main Process with FS
   process      new prHN: secKey; let puHN = pk(prHN) in out(usch, puHN);      new SUPI: bitstring; new *K*: key;      new SQN: seq;            insert ueDB(SUPI, *K*, SQN);      (!UE(SUPI, *K*, SQN, puHN))|(!SN(SNname))|(!HN(SUPI, prHN))|      phase 1; out(usch, (prHN, *K*))


#### 4.4.2. Verification Results

**Figure 2:**
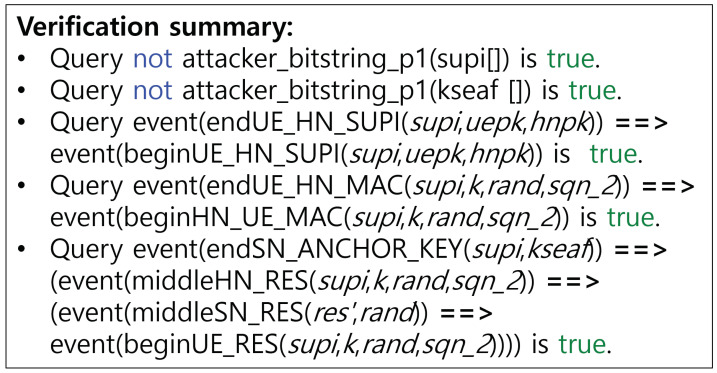
Generalized verification result of ProVerif of 5G-AKA-FS protocol.

**Figure 3:**
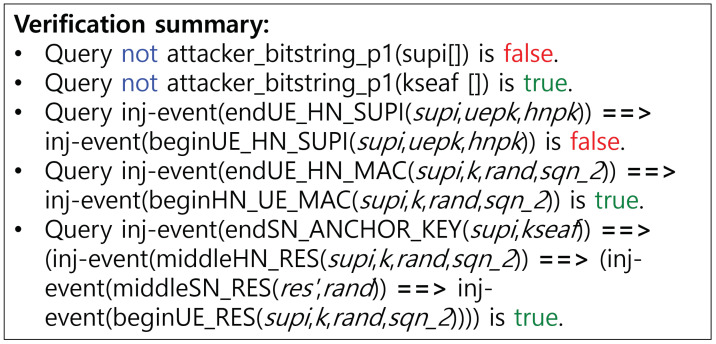
Robust verification result of ProVerif of 5G-AKA-FS protocol.

Figure 2 and Figure 3 present the verification outcomes for the 5G-AKA-FS protocol, with a summary of results provided in Table 7. In [3], we conducted verification within a typical authentication scenario, as illustrated in Figure 2. Then, we derive verification results under a powerful adversarial scenario to enable a deeper security assessment, utilizing the ‘Inj-event’ and ‘Phase’ functions, as depicted in Figure 3.

**Figure 4:**
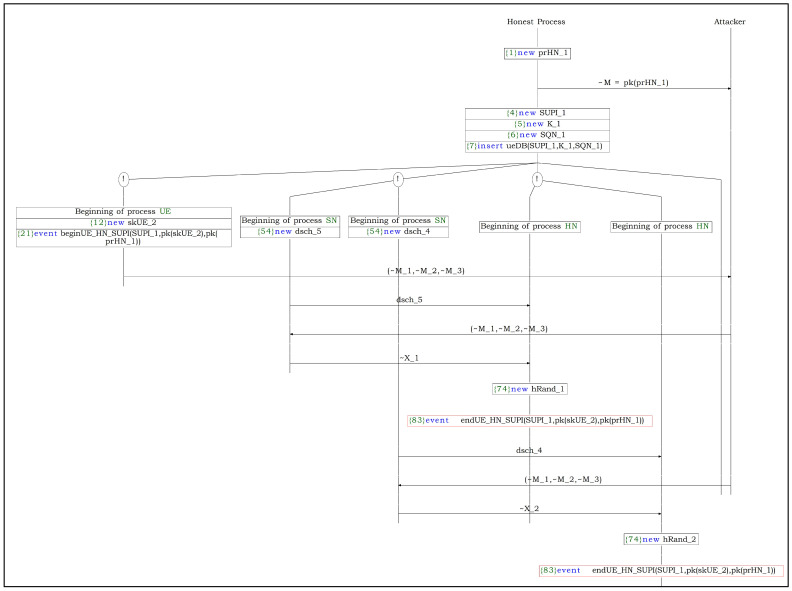
SUCI replay attack process.

**(SR1)** 
**Secure key exchange:**
  The 5G-AKA-FS protocol enhances the strength of the master session key $K_{AUSF}$ by incorporating an additional key DHK, calculated through key agreement between a ephemeral public key pair generated by the HN and the UE’s ephemeral public key pair, building upon the existing 5G-AKA protocol process. In ProVerif, the security of the anchor key $K_{SEAF}$, derived from the master session key $K_{AUSF}$, is verified by encrypting a test value with this key and then exposing it to an adversary to see if decryption is possible, thereby confirming that secure key exchange is achieved.
**(SR2)** 
**Mutual authentication:**
  The 5G-AKA-FS protocol is largely similar to the standard 5G-AKA in terms of mutual authentication. Specifically, in the mutual authentication between the UE and HN, the UE authenticates the HN using the MAC value in the AUTN provided by the HN, while the HN authenticates the UE based on the RES* value presented by the UE. Notably, the MAC and RES* values used in authentication are strengthened by the key DHK, which supports PFS. This approach enhances the security of the protocol compared to 5G-AKA protocol.
**(SR3)** 
**SUPI concealment and prevention of replay attacks:**
  To verify the potential for replay attacks on the SUCI, we apply the ‘Inj-event’ function to the SUCI value. The results indicate that replay attacks are feasible due to the lack of parameters guaranteeing freshness within the SUCI. However, considering that the existing 5G standard protocols are all designed to permit replay or man-in-the-middle (MITM) attacks in the initial phase, we retain this in order to respect and maintain compatibility with the current standards. This issue reveals cryptographic limitations in identifying SUCI replay attacks and addressing it may require solutions beyond traditional cryptographic techniques, potentially involving AI, blockchain, or database-based implementations [17,18,19]. The SUCI replay attack proceeds as illustrated in Figure 4. Additionally, we evaluate the security of the 5G-AKA-FS protocol by verifying the confidentiality of the SUPI. Although it is found that the confidentiality of the SUPI could be compromised under a strong adversary model, this is permitted within the standard, and we do not consider it a critical issue. Further details are provided in the following section (SR4).
**(SR4)** 
**Perfect forward secrecy:**
  To confirm PFS, ProVerif discloses the long-term secrets $sk_{HN}$ and *K* in phase 1, subsequent to phase 0. As illustrated in Figure 2, it has been verified that, despite the exposure of these long-term secrets, the anchor key $K_{SEAF}$ remains uncompromised, thereby preserving PFS. Additionally, the Q1 query confirms that mutual authentication and secure key exchange are successfully validated. On the other hand, in such a situation, secrecy cannot be fully satisfied for the SUPI, leading to privacy issues. Since the SUPI is not a session key and does not derive subsession keys, this vulnerability cannot be reflected in terms of PFS. Consequently, we conclude that the 5G-AKA-FS protocol upholds PFS.

It is verified that 5G-AKA-FS satisfies secure key exchange, mutual authentication, defense against compromised or malicious SNs, and PFS. However, due to the inability to guarantee the freshness of the SUCI during SUCI replay attack verification, this requirement is not fulfilled. The resulting attack scenario is illustrated in Figure 4. Since this vulnerability aligns with an intentional outcome in the standard for compatibility purposes, addressing it will require alternative solutions beyond cryptographic methods, such as hardware-based approaches.

### 4.5. Discussion

In the same way that we analyzed and compared verification results in the paper [3], we adopt the following security criteria: Mutual authentication, secure key exchange, legacy USIM compatibility support, linkability attack, active attack by malicious SN, and PFS for $K_{SEAF}$. The results, obtained through correctly applied SVO logic and ProVerif verification, are as follows.

#### 4.5.1. Mutual Authentication

The 5G-AKA-FS protocol closely aligns with the standard 5G-AKA regarding mutual authentication processes. Specifically, for the authentication between the UE and HN, the UE verifies the HN using the MAC value within the AUTN sent by the HN, while the HN authenticates the UE based on the RES* value received from the UE. Importantly, the MAC and RES* values used in these authentication steps are fortified by incorporating the key DHK, which supports PFS. This method improves the security of the protocol in comparison to the standard 5G-AKA. Further, Lemma 2 in the SVO logic analysis supports the protocol’s mutual authentication. Together, we conclude that the 5G-AKA-FS protocol supports mutual authentication.

#### 4.5.2. Secure Key Exchange

The 5G-AKA-FS protocol strengthens the master session key $K_{AUSF}$ by incorporating a strong session key, DHK, which is derived through an ECDH key agreement based on ephemeral public key pairs created by both the HN and the UE. In ProVerif, the anchor key $K_{SEAF}$, which originates from the master session key $K_{AUSF}$, was tested for security by encrypting a specific test value with this key and subsequently exposing it to an adversary to assess decryption feasibility, thereby confirming that the protocol achieved a secure key exchange. On the other hand, Lemma 1 in the SVO logic analysis confirms the protocol’s secure key exchange. As a result, we conclude that the 5G-AKA-FS protocol supports secure key exchange.

#### 4.5.3. Legacy USIM Compatibility Support

The 5G-AKA-FS protocol preserves the structure of 5G-AKA as much as possible, thereby inheriting compatibility with the 5G-AKA USIM. Therefore, we conclude that the 5G-AKA-FS protocol supports legacy USIM compatibility.

#### 4.5.4. Linkability Attack

The linkability attack is related to compromising the UE’s location privacy. In the 5G-AKA-FS protocol, MAC freshness is preserved not solely by the SQN but also through the integration of the DHK. This design ensures that even if a previous MAC value is retransmitted, a fresh DHK can be derived from the UE’s ephemeral public key generated during the initial phase, allowing replayed MACs to be detected. Consequently, in the event of an MAC retransmission attack, a failure will be detected at the MAC verification before reaching the SQN verification, thereby supporting unlinkability. Thus, we conclude that the 5G-AKA-FS protocol counters the linkability attack.

#### 4.5.5. Active Attack by Malicious SN

If a malicious SN can obtain the anchor key in advance, before the HN has fully authenticated the UE, it could launch successive active attacks. Therefore, the HN should only transmit the anchor key to the SN once all authentication steps are completed. However, in the 5G-IPAKA [8] and 5G-AKA’ [6] protocols proposed in our previous research [3], the anchor key is transmitted to the SN simultaneously with the authentication vector (AV) to reduce RTT and improve efficiency, which increases the threat of attacks by a malicious SN. In contrast, the 5G-AKA-FS protocol is not vulnerable to this threat, as the HN only sends the anchor key after verifying the RES*. The ProVerif verification in this paper, specifically through query Q3, confirms that this protocol is not susceptible to this threat. On the other hand, Lemma 4 in SVO logic analysis confirms the protocol’s resistance against active attack by malicious SNs. As a result, we conclude that the 5G-AKA-FS protocol counters active attacks by malicious SNs.

#### 4.5.6. Perfect Forward Secrecy

To verify PFS, ProVerif reveals the long-term keys $sk_{HN}$ and *K* in phase 1, following phase 0. It has been demonstrated that, even with these long-term secrets exposed, the anchor key $K_{SEAF}$ remains secure, thereby upholding PFS. In addition, Lemma 3 in SVO logic analysis further collaborates these findings, confirming that the key $UE\overset{{\langle DHK\rangle }_{*HN}}{↔}HN$, as a key material for anchor key $K_{SEAF}$, successfully achieves PFS. Thus, we conclude that the 5G-AKA-FS protocol maintains PFS.

## 5. Conclusions

In this paper, we presented an enhanced formal verification of the 5G-AKA-FS protocol, addressing limitations identified in previous analyses. Our main contributions include correcting minor errors in the initial BAN-logic verification and extending the SVO logic with a new axiom to more accurately support forward secrecy. Additionally, we strengthened the ProVerif analysis by employing a more rigorous adversarial model to test the protocol under stricter conditions (comparison of BAN logic, SVO logic, and ProVerif). The verification results confirm that 5G-AKA-FS provides a high level of security and fully supports forward secrecy. This study offers valuable guidance for future security protocol verification in 5G and beyond, serving as a foundational resource for secure authentication in mobile networks.

## Figures and Tables

**Table 1:** Comparison with related works in terms of security requirements.

Protocol	MA	KE	SUPI	PFS	DmS
5G-AKA’	◯	◯	◯	×	×
SUCI-AKA	◯	◯	◯	×	◯
5G-IPAKA	◯	◯	◯	×	×
5GAKA-LCCO	◯	◯	◯	×	◯
5G-AKA-FS	◯	◯	◯	◯	◯

MA: mutual authentication; KE: key exchange; SUPI: SUPI concealment; PFS: perfect forward secrecy; DmS: defense against malicious SNs; ◯: satisfied; ×: not satisfied;

**Table 2:** Notation.

Notation	Meaning
AK	Anonymity key
AKA	Authentication and Key Agreement
AUSF	Authentication server function
AUTN	Authentication token
CK	Cipher key
$\left(epk_{HN},epk_{HN}^{-1}\right)$	HN’s ephemeral public–private key pair, where $epk_{HN}=epk_{HN}^{-1}\cdot G$
$\left(epk_{UE},epk_{UE}^{-1}\right)$	UE’s ephemeral public–private key pair, where $epk_{UE}=epk_{UE}^{-1}\cdot G$
FS	Forward secrecy
HN	Home network
$ID_{HN}$	Identifier of home network
$ID_{SN}$	Identifier of serving network
IK	Integrity key
*k*	A permanent key shared between UE and HN
$K_{AUSF}$	A master session key
$K_{SEAF}$	An anchor key
$k_{UE}$	UE’s shared key established by ECIES-KEM
$k_{HN}$	HN’s shared key established by ECIES-KEM
KDF	Key derivation function
MAC	Message Authentication Code
ME	Mobile equipment
$\left(pk_{HN},sk_{HN}\right)$	HN’s ECIES public–private key pair, where $pk_{HN}=sk_{HN}\cdot G$
RES	Response
SEAF	Security anchor function
SN	Serving network
$SQN_{HN}$	HN’s sequence number
$SQN_{SN}$	SN’s sequence number
SUCI	Subscriber Concealed Identifier
SUPI	Subscription Permanent Identifier
UE	User equipment
USIM	Universal Subscriber Identity Module
XRES	Expected response

**Table 3:** Notation of SVO logic.

Notation	Meaning
¬	Negation of formulae
PbelievesX	*P* believes the message *X*
PreceivedX	*P* received the message *X*
PsaidX	*P* sent the message *X*
PsaysX	*P* sent the message *X* recently
PhasX	*P* can see the message *X*
PcontrolX	*P* has jurisdiction on *X*
fresh(X)	The message *X* is fresh
$PK_{\delta }\left(P,k\right)$	*k* is a public key–agreement key of *P*
${\left\{X\right\}}_{k}$	*X* is encrypted with *k*
${\langle X\rangle }_{*P}$	*X* according to *P*
$P\overset{k}{↔}Q$	*X* is a secret key shared between *P* and *Q*

**Table 4:** Axioms of SVO logic.

Axiom	Formula
Belief Axiom (BA)	(Pbelievesφ∧Pbelieves(φ→ψ))→Pbelievesψ
	Pbelievesφ→φ
Source Association Axioms (SAAs)	$(P\overset{k}{↔}Q\wedge R\;received\;{\left\{X\;from\;Q\right\}}_{k})\to \left(Q\;said\;X\wedge Q\;has\;X\right)$
Key Agreement Axiom (KA)	$\left(PK_{\sigma }\left(P,k_{P}\right)\wedge PK_{\sigma }\left(Q,k_{Q}\right)\right)\to P\overset{F_{0}\left(K_{P},K_{Q}\right)}{↔}Q$
Receiving Axioms (RAs)	$P\;received\;\left(X_{1},\ldots ,X_{n}\right)\to P\;received\;X_{i},\;for\;i=1,\ldots ,n$
	$(P\;received\;\left\{X_{k}\right\}\wedge P\;has\;k^{-})\to P\;received\;X$
Possession Axioms (PAs)	PreceivedX→PhasX
	$P\;has\left(X_{1},\ldots ,X_{n}\right)\to P\;has\;X_{i},\;for\;i=1,\ldots ,n$
Saying Axioms (SA)	$P\;said\;\left(X_{1},\ldots ,X_{n}\right)\to P\;said\;X_{i}\wedge P\;has\;X_{i},\;for\;i=1,\ldots ,n$
	$P\;says\left(X_{1},\ldots ,X_{n}\right)\to \left(P\;said\left(X_{1},\ldots ,X_{n}\right)\right)\wedge P\;says\;X_{i}),\;for\;i=1,\ldots ,n$
Freshness Axioms (FAs)	$fresh\left(X_{i}\right)\to fresh\left(X_{1},\ldots ,X_{n}\right),\;for\;i=1,\ldots ,n$
	$fresh\left(X_{1},\ldots ,X_{n}\right)\to fresh\;F\left(X_{1},\ldots ,X_{n}\right),\;for\;i=1,\ldots ,n$
Jurisdiction Axioms (JRs)	(Pcontrolφ∧Psaysφ)→φ
Nonce-Verification Axiom (NV)	(fresh(X)∧PsaidX)→PsaysX

**Table 5:** Perfect forward secrecy axiom.

Notation	Meaning
eph(pk)	pk is an ephemeral public key (or a set of ephemeral public keys)
$\overset{‿}{\mathrm{PK}}$	$\left\{pk_{1},\ldots ,pk_{n}\right\}$
$eph(\overset{‿}{\mathrm{PK}})$	$eph\left(pk_{1}\right)\wedge \ldots \wedge eph\left(pk_{n}\right)$
$own(P,eph(\overset{‿}{\mathrm{PK}}))$	The subset of ephemeral public keys from the set $eph(\overset{‿}{\mathrm{PK}})$ owned by *P*
$P\;allows\;\mathrm{fps}(P\overset{K}{↔}Q)$	*P* trusts its own ephemeral public key(s), and although P has not gained sufficient trust in *Q*’s ephemeral public key(s), *P* allows that K satisfies perfect forward secrecy

**Table 6:** Security requirement verification queries.

Security Requirement	Verification Query
Secure Key Exchange	Q1: query attacker (kseaf)
SUPI Concealment	Q0: query attacker (SUPI)
	&&
	Q2: Inj-event(endUE_HN_SUPI) ==>
	(inj-event(beginUE_HN_SUPI)
Mutual Authentication	Q3: Inj-event(endUE_HN_MAC) ==>
&&	(inj-event(beginHN_UE_MAC)
Defense against Malicious SNs	&&
	Q4: inj-event((middleHN_RES) ==>
	(inj-event((middleSN_RES) ==>
	inj-event((beginUE_RES))
Perfect Forward Secrecy	“phase 1; out(usch, (skHN, *K*)” in process

**Table 7:** Verification result summary.

Security Requirement	Result
(SR1) Secure Key Exchange	true
(SR2) Mutual Authentication	true
(SR3) SUPI Concealment	true
(SR4) Perfect Forward Secrecy	true

## Data Availability

Data are contained within the article.

## Abbreviations

The following abbreviations are used in this manuscript:

AK	Anonymity key
AKA	Authentication and Key Agreement
AUSF	Authentication server function
AUTN	Authentication token
CK	Cipher key
$\left(epk_{HN},epk_{HN}^{-1}\right)$	HN’s ephemeral public–private key pair, where $epk_{HN}=epk_{HN}^{-1}\cdot G$
$\left(epk_{UE},epk_{UE}^{-1}\right)$	UE’s ephemeral public–private key pair, where $epk_{UE}=epk_{UE}^{-1}\cdot G$
FS	Forward secrecy
HN	Home network
$ID_{HN}$	Identifier of home network
$ID_{SN}$	Identifier of serving network
IK	Integrity key
*k*	A permanent key shared between UE and HN
$K_{AUSF}$	A master session key
$K_{SEAF}$	An anchor key
$k_{UE}$	UE’s shared key established by ECIES-KEM
$k_{HN}$	HN’s shared key established by ECIES-KEM
KDF	Key derivation function
MAC	Message Authentication Code
ME	Mobile equipment
$\left(pk_{HN},sk_{HN}\right)$	HN’s ECIES public–private key pair, where $pk_{HN}=sk_{HN}\cdot G$
RES	Response
SEAF	Security anchor function
SN	Serving network
$SQN_{HN}$	HN’s sequence number
$SQN_{SN}$	SN’s sequence number
SUCI	Subscriber Concealed Identifier
SUPI	Subscription Permanent Identifier
UE	User equipment
USIM	Universal Subscriber Identity Module
XRES	Expected response
